# A Hybrid Skin Cyst at an Unusual Location: A Case Report

**DOI:** 10.7759/cureus.68378

**Published:** 2024-09-01

**Authors:** Maria Koleva, Tomo Lisner, Angelina Mollova, Petar-Preslav Petrov, Dorian Dikov

**Affiliations:** 1 Department of General and Clinical Pathology, Medical University of Plovdiv, Plovdiv, BGR; 2 Department of Pathology, Jossigny Hospital, Jossigny, FRA; 3 Department of Anatomy, Histology and Embryology, Medical University of Plovdiv, Plovdiv, BGR

**Keywords:** benign cutaneous cyst, pilomatricoma, trichilemmal cyst, epidermal cyst, hybrid cyst

## Abstract

Hybrid cysts originate from more than two components of the pilosebaceous unit. The pathogenesis of this cystic lesion remains unclear. Most of the investigated cases have scalp and face predilection. The lesion is predominantly observed in females. We present a case of a 69-year-old woman with a painless cutaneous nodule measuring 0.7 cm on the right calf. After a surgical excision, the histological investigation showed that the normal epidermis was elevated by a deeply encapsulated intradermal cyst, which was lined by two distinct histological types of epithelium with an abrupt transition between them. One-half of the cystic surface consisted of a keratinizing squamous layer with a granular layer and keratin lamellae, while the other half was lined by epithelium showing pilomatricial differentiation. Finally, an extremely rare diagnosis was set of a hybrid cyst.

## Introduction

The pilosebaceous unit can give rise to a variety of cutaneous cystic lesions - epidermal, trichilemmal cysts, steatocystomas, or pilomatricomas [1]. They all have clinical similarities and can only be differentiated by their histological features [2,3]. Epidermal cysts, which originate from the follicular infundibulum, are lined with stratified squamous epithelium, while trichilemmal cysts exhibit keratinization similar to that seen in the isthmus of the hair follicle’s outer root sheath. Pilomatricomas are benign tumors derived from hair matrix cells and consist of basophilic and shadow cells. A hybrid cyst is a very rare lesion. It was first described by McGavran et al. in 1966 as a cystic tumor combining infundibular and trichilemmal cysts. Brownstein et al. later reported seven cases of hybrid cysts, where the upper part near the epidermis was presented by an infundibular cyst and the deeper part was presented by a trichilemmal cyst. Subsequently, Requena et al. proposed that a hybrid cyst could include any combination of cysts originating from the pilosebaceous unit [2]. Its pathogenesis has not been fully established [3]. It presents as a cyst, typically composed of more than two components of the pilosebaceous unit [1,3]. Most cases show a female predominance, with a preference for the scalp and face. While different histological combinations can occur, the most common is a mix of infundibular and trichilemmal cysts (60%) [2]. We present a case of a hybrid cyst of epidermal and pilomatricial type that developed from the infundibular and matrix parts of the hair follicle at an unusual location on the lower limb of a 69-year-old female patient. It is our aim to attract attention toward an uncommon variation of an already exceedingly rare lesion and explore its clinical significance. A correct diagnosis is required because these skin cystic proliferations are observed in half of patients with Gardner syndrome, an autosomal dominant hereditary syndrome [2].

## Case presentation

**Figure 1:**
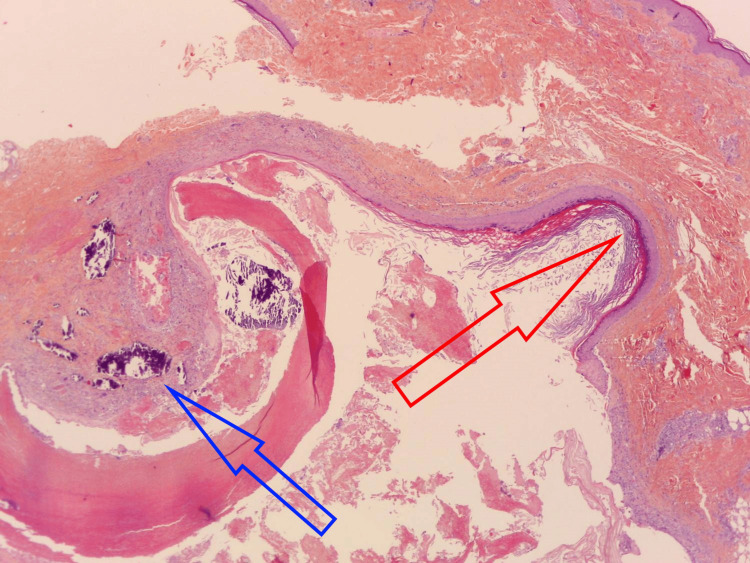
The cyst is composed of two distinct components: the epidermal component on the right side (red) and the pilomatricial component on the left side (blue) of the picture. Hematoxylin-eosin-saffron, 50x.

A 69-year-old woman with no prior relevant medical history presented with a painless cutaneous nodule on her right calf. A total excision of the lesion was performed. Macroscopic examination showed an indurated cyst measuring 0.7 cm on the long axis, well-defined, and containing white lumpy material. Histologically, the normal epidermis was elevated by a deep encapsulated intradermal cystic formation lined by two very distinct histological types of epithelium with a sudden transition (Figure 1).

**Figure 2:**
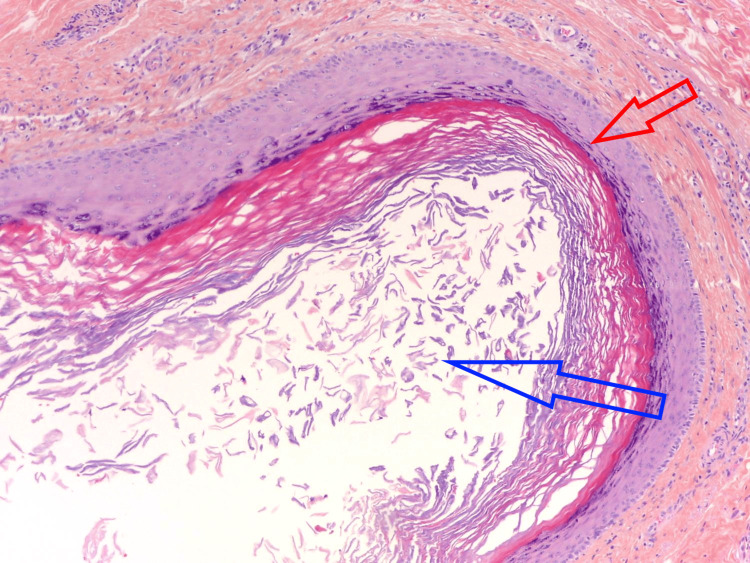
Epidermal component of the cyst. The lining contains a granular layer (red) and lamellated keratin in the lumen (blue). Hematoxylin-eosin-saffron, 100x.

**Figure 3:**
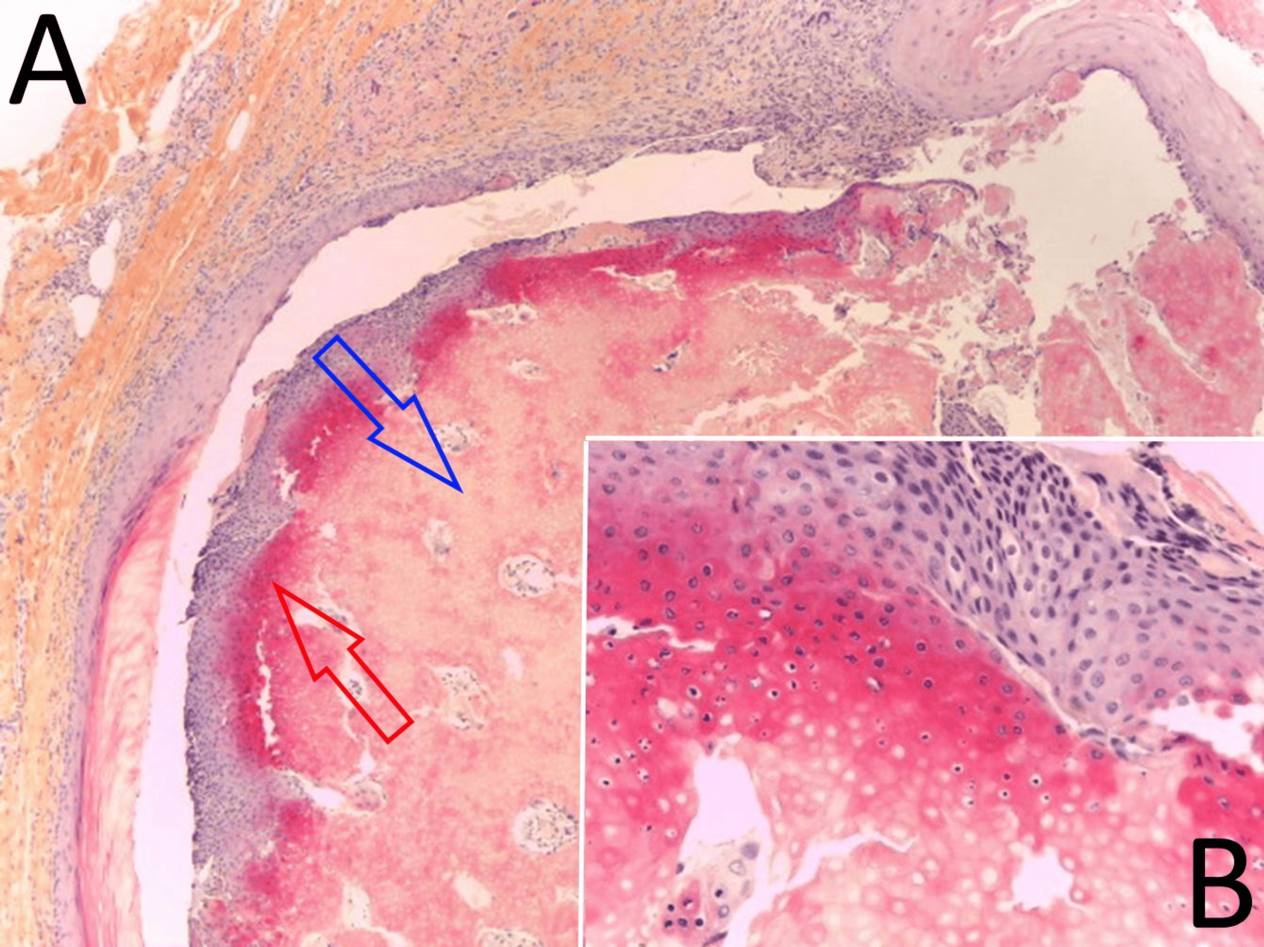
Pilomatricial component of the hybrid cyst. (A) Hematoxylin-eosin-saffron, 50x. A nest of small basaloid cells (red) and eosinophilic ghost cells (blue) with a relatively gradual transition are observed. (B) Hematoxylin-eosin-saffron, 400x. The transition between the two types of cells is seen.

Half of the cystic surface was composed of a keratinizing squamous coating, with a granular layer and lamellae of keratin (Figure 2). The other half was covered by epithelium with pilomatricial differentiation, consisting of clusters of small basaloid cells and eosinophilic ghost cells with a more or less gradual transition between these two cell types (Figure 3). 

**Figure 4:**
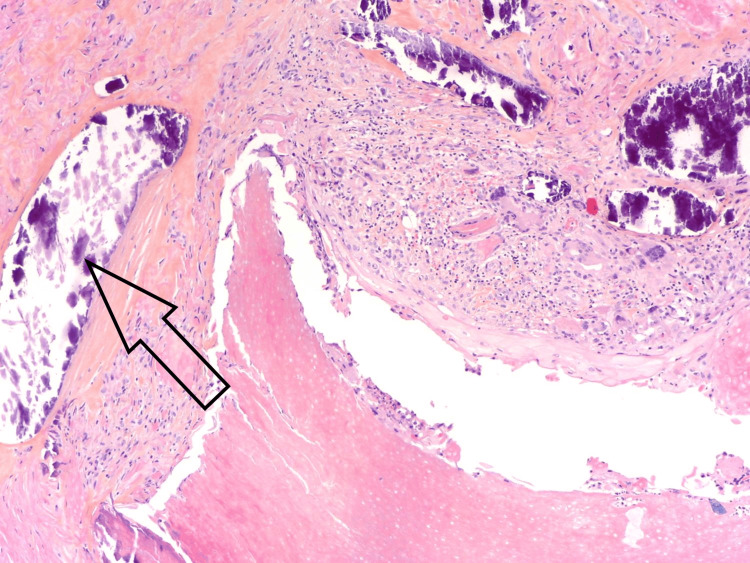
Granulomatous inflammation with multinucleated giant cells in contact with ghost cells and calcifications (arrow). Hematoxylin-eosin-saffron, 100x.

There was a foreign body type granulomatous inflammatory reaction in contact with the mummified epithelial trabeculae, containing some calcifications (Figure 4).

Based on the aforementioned characteristics of the lesion, a conclusive diagnosis was made - a hybrid follicular cyst of epidermal and pilomatricial type. As the total resection of the cyst was successfully carried out, the patient was discharged without further complaints or clinical manifestations. Her follow-up checks over the course of a year revealed the presence of no new cysts and a lack of complications.

## Discussion

The hybrid follicular cyst is an association between two or more cystic components that can develop from different parts of the pilosebaceous unit [1]. Thus, it is a cutaneous cyst exhibiting epidermal, trichilemmal, or pilomatricial type of keratinization; the first two in combination are the most frequently occurring (60% of reported cases) [2].

The hybrid cyst of epidermal and pilomatricial type, such as in the case presented by us, developing from the infundibular and matrix parts of the hair follicle is a rare entity [3]. Around 40 cases are described in the literature as per our knowledge [3]. It represents approximately 10% of hybrid cutaneous cysts, affecting predominantly women [3]. In a case series study conducted by Kim et al., the lesion is found in individuals from 11 to 50 years of age, the average age being 27 years; it is more frequently located in the upper part of the body [3]. The cyst in our patient appeared at an older age (69 years) and was located on the lower limb, which are less commonly reported features of this lesion.

Macroscopically, the hybrid epidermal and pilomatricial cyst is not well described in the clinical literature. Our case presented a cyst of 0.7 cm in diameter that was firm upon palpation with white-gray content, consisting of a macroscopic mixture of the epidermal cyst and the pilomatricoma.

The diagnosis of the hybrid cyst of epidermal and pilomatricial type is histological, based on the association of two types of keratinization of the border of the cyst: the epidermal type with a keratinizing squamous layer, provided with a granular layer with lamellae and keratin in the center, and the pilomatricial type, made up of nests of small basaloid cells with particular maturation and sheets of ghost cells [4]. There are often calcifications and ossifications within the ghost cell areas, with a foreign body type granulomatous inflammatory reaction upon contact [4]. The transition between the two types of keratinization is abrupt. Immunohistochemistry is not useful for this diagnosis [4].

Skin cystic proliferations are observed in 53% of patients with Gardner syndrome, an autosomal dominant hereditary syndrome [4]. This is a phenotypic expression of familial adenomatous polyposis associated with colorectal adenomas and adenocarcinomas before the age of 40, on one hand, and on the other hand, extra colorectal manifestations such as osteomas, gastroduodenal polyps, and skin lesions such as epidermoid cysts or multiple pilomatricomas occurring in childhood [4]. It should be noted that 37% of skin cysts in patients affected by this syndrome present hybrid epidermal and pilomatricial characteristics [5]. At our present level of understanding, the quantity of the cysts (i.e., the presence of multiple cysts as opposed to fewer lesions) and the quality of the cysts (i.e., their hybrid nature) are equally important clinical markers for association with the presence of Gardner syndrome [4,5]. We emphasize the need for further examination as to which of these two characteristics is more indicative of the presence of the aforementioned disease.

In our case, the patient had neither a family history nor clinical signs suggesting Gardner syndrome. Gastro- and colonoscopy carried out after our diagnosis did not find any polyps. In conclusion, the hybrid epidermal and pilomatricial cyst is a curious, rare, benign lesion. The correct diagnosis of such cysts is important because it can reveal a hereditary disease predisposing to cancer.

## Conclusions

We have presented a clinical case of a hybrid cyst with a combination of epidermal and pilomatricial differentiation of the epithelium. We emphasize the age of the patient (outside of the usual range for the formation of such cysts) and the unusual location of the lesion (the lower limb) and demonstrate that hybrid cysts can appear outside of expected circumstances and in otherwise healthy individuals not suffering from Gardner syndrome or predisposed to colorectal malignancies and pathologies. The patient we monitored showed no additional complications related to the hybrid cyst. She remains in good health, but regular follow-up visits have been recommended.

## References

[1] Requena L, Sánchez Yus E (1991). Follicular hybrid cysts: an expanded spectrum. Am J Dermatopathol.

[2] Takeda H, Miura A, Katagata Y, Mitsuhashi Y, Kondo S (2003). Hybrid cyst: case reports and review of 15 cases in Japan. J Eur Acad Dermatol Venereol.

[3] Kim MS, Lee JH, Son SJ (2012). Hybrid cysts: a clinicopathological study of seven cases. Australas J Dermatol.

[4] Cribier B (2009). Accurate diagnosis of adnexal tumors can be a matter of life or death (Article in French). Ann Dermatol Venereol.

[5] Cooper PH, Fechner RE (1983). Pilomatricoma-like changes in the epidermal cysts of Gardner's syndrome. J Am Acad Dermatol.

